# English Flipped Classroom Teaching Mode Based on Emotion Recognition Technology

**DOI:** 10.3389/fpsyg.2022.945273

**Published:** 2022-07-13

**Authors:** Lin Lai

**Affiliations:** School of Foreign Languages, Xihua University, Chengdu, China

**Keywords:** speech emotion recognition, image emotion recognition, English flipped classroom, SVM algorithm, feature extraction

## Abstract

With the development of modern information technology, the flipped classroom teaching mode came into being. It has gradually become one of the hotspots of contemporary educational circles and has been applied to various disciplines at the same time. The domestic research on the flipped classroom teaching mode is still in the exploratory stage. The application of flipped classroom teaching mode is still in the exploratory stage. It also has many problems, such as low class efficiency, poor teacher-student interaction, outdated teaching modes, not student-centered, etc., which lead to poor students’ enthusiasm for learning. Therefore, the current English flipped classroom teaching mode still needs to be tested and revised in practice. Combined with emotion recognition technology, this paper analyzes speech emotion recognition, image emotion recognition, and audition emotion recognition technology and conducts a revision test for the current English flipped classroom teaching mode. It uses the SVM algorithm for one-to-one method and dimension discretization for emotion recognition, and finds that the recognition results after different dimension classification recognition are improved for each emotion. Among them, the recognition rate of different dimension classification recognition methods is 2.6% higher than that of one-to-one method. This shows that under the same conditions, the emotion recognition technology of different dimension classification recognition methods is higher.

## Introduction

With the continuous innovation of educational information, the teaching model is catering to the information age. The flipped classroom comes with the development of education reform, and it is a “top-down” development model. In the information age, the concept of education and teaching mode also has new requirements, and it will also face new challenges. For the problems of outdated teaching mode and the failure of teaching concept to keep up with the development of the times, this paper constructs and optimizes the teaching mode of English flipped classroom by conducting targeted research on speech emotion recognition, image emotion recognition, and audition fusion emotion recognition technology in the teaching mode of flipped classroom.

Emotion recognition technology is the main component in the field of emotion feature computing. Mohammed and Karim (2020) proposed a new method for facial emotion recognition based on graph mining to change the representation of face regions. He represented face regions as node and edge graphs and used the gSpan frequent subgraph mining algorithm to find frequent substructures in a graph database for each emotion. He conducted different experiments using the Surrey Audio-Visual Expression of Emotion (SAVEE) database, and the final system was 90.00% accurate. The results show a significant improvement in the accuracy of the system compared to the currently published work in the SAVEE database. Jacob (2017) compared two speech emotion recognition models based on vocal tract features, namely, the first four formants and their respective bandwidths. The first model is based on decision trees and the second model uses logistic regression. While decision tree models are based on machine learning, regression models have a strong statistical foundation. The logistic regression model and decision tree model developed for several binary classification situations are validated through speech emotion recognition experiments on the Malayalam emotional speech database. Gomathy (2021) believed that human emotion recognition is possible using speech signals. Speech emotion recognition is a critical and challenging task, in which feature extraction plays a crucial role in its performance. Determining affective states in speech signals is a very challenging area for many reasons. The first problem of all speech emotion systems is to select the best features that are sufficient to distinguish various emotions. In response to these problems, he proposed an Enhanced Cat Swarm Optimization (ECSO) algorithm for feature extraction. This algorithm is not used in any existing speech emotion recognition methods. The method achieved excellent performance in terms of accuracy, recognition rate, sensitivity, and specificity. Andy and Kumar (2020) believed that speech, as a means of biometric measurement through use and meaning, has become an important part of speech development. They attempt to explain various speech and emotion recognition techniques, as well as a comparison between several approaches based on existing algorithms and primarily speech-based approaches. Therefore, they enumerate and differentiate the specification, database, classification, feature extraction, enhancement, segmentation, and processing techniques for speech emotion recognition.

With the continuous advancement of education informatization, the teaching mode is also constantly updated. The development model of the flipped classroom has been paid increasingly attention by scholars. Chang (2021) proposed a flipped classroom teaching model for college English based on big data and deep neural networks. They selected 230 students from two classes in the second grade of a college English major as the research object. As for the weight of deep neural network prediction and effective analysis of influencing factors, it has certain reference significance for the optimization of flipped classroom teaching mode. Feng (2017) believed that as a new educational technology, the flipped classroom teaching mode of college English based on MOOC effectively promotes the improvement of classroom teaching effect and teaching objectives. It can promote students’ communicative competence and learning ability, and at the same time realize personalized learning and improve learning ability. Starting from the reality of college English branch courses, this paper tries to apply this model to college English teaching. He proposed the idea of introducing the MOOC-based flipped classroom in college English courses into English courses and studied the effect of college English teaching based on the MOOC flipped classroom model from a quantitative point of view. Zhou and Zhang (2017) believed that learners face two major problems: information overload and knowledge fragmentation. In this context, university teaching is no longer limited to the traditional classroom model. Instead, integrating MOOC, SPOC, and flipped classroom models is a new trend. He combined mobile Internet technology with classroom teaching and teaching materials to build a flipped English classroom teaching model to solve the problem of disconnection between teaching software and teaching materials in the teaching process. It comprehensively improves the level of language learning in time and space, and truly realizes borderless learning.

The above literature have detailed and comprehensive research on emotion recognition technology and flipped classrooms, which has guiding significance for the description below. It studies the influence of emotion recognition technology in the teaching mode of English flipped classroom. It also conducts targeted research on speech emotion recognition, image emotion recognition, and audition fusion emotion recognition technology in the English flipped classroom teaching process. Constructing and optimizing the English flipped classroom teaching mode is conducive to optimizing teaching resources and improving the interaction between students and teachers. This is conducive to educators to formulate a teaching model tailored to local conditions, improve teaching efficiency, and make the flipped classroom get the best results. In the actual application process, it is found that the speech recognition rate of the dimension classification recognition model is 2.6% higher than that of the one-to-one model. In the emotion recognition experiment of decision-level fusion of different strategies, the product rule has the highest recognition rate of 92.27%. Compared to summation, it finds that the mean rule fusion method is 2.39% and 1.59% higher, respectively. In the RNN emotion recognition and feature fusion recognition experiments, the decision layer product rule recognition rate is the highest, reaching 92.27%. After conducting sentiment analysis on the flipped classroom teaching model, it conducts a comparative analysis of student performance. It is found that after the improvement of the teaching mode, the students’ enthusiasm for learning is improved, and the performance is also improved accordingly.

## English Flipped Classroom Teaching Mode Method Based on Emotion Recognition Technology

### Speech Emotion Recognition

**Figure 1:**
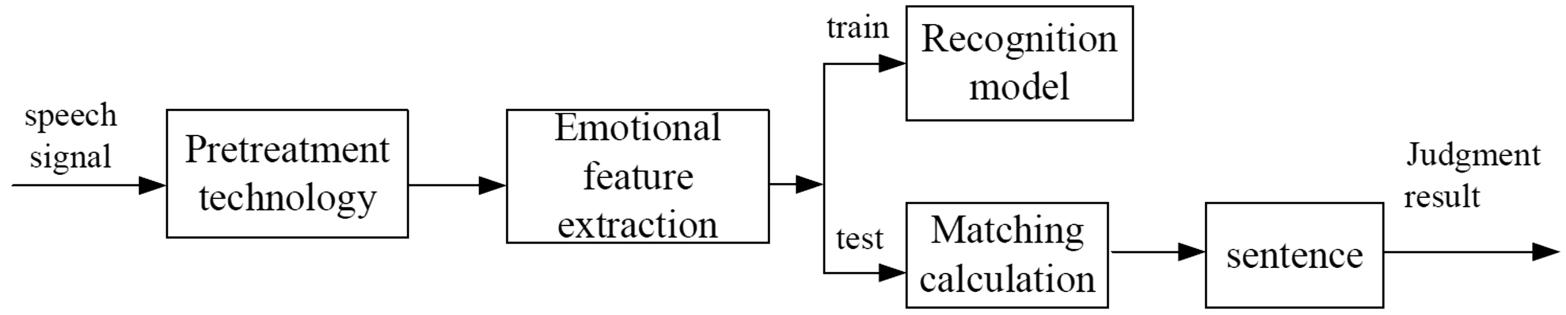
Block diagram of speech emotion recognition system.

Speech emotion recognition technology is to use computer technology to analyze the emotion of speech signal. It extracts, identifies, and classifies valuable emotional features, and builds models with corresponding parameters. The updated parameters can then be trained and matched by computer means to obtain the corresponding recognition results (Poorna and Nair, 2019). Different from speech recognition, speech emotion recognition utilizes the personality characteristics of different emotional states. It removes the interference of textual information as much as possible and emphasizes the personality characteristics of different emotional states. The speech recognition mainly emphasizes the text information of the speech signal. It does not consider the emotional information of the speaker, as long as it recognizes the content of the speaker’s speech. Figure 1 shows the block diagram of the speech emotion recognition system. A complete speech emotion recognition system mainly includes speech signal preprocessing, feature parameter extraction, model training, pattern matching, and judgment. Speech emotion recognition is the key to human-computer emotional interaction. Effective recognition of speech emotion can improve speech intelligibility. It enables all kinds of smart devices to understand the user’s intentions to the maximum extent and improves the level of robot humanization, to better serve human beings.

#### Overview of Speech Emotional Features

Speech emotion features mainly include speech rate, speech fundamental frequency, and speech energy. Speech rate is a relatively intuitive feature of speech emotion feature analysis. Speech rate not only reflects the speed of speaking, but also reflects the emotional changes when speaking. It is usually measured by the number of syllables. The fundamental frequency of speech is closely related to the vocal cords, which can be analyzed according to the acoustic characteristics of speech. It is mainly manifested in that people’s voice is elongated and thickened, which is mainly caused by the change of air pressure between the glottis. The fundamental frequency can well reflect the activation value of voice emotion, so the fundamental frequency is widely used as an important feature in speech emotion recognition. The energy in speech can effectively reflect the pitch of the sound, which reflects the change of the amplitude of the speech signal with time. The pitch of the voice can intuitively see changes in a person’s emotional characteristics, such as changes in emotions such as anger, sadness, and happiness. The energy in the speech is also different, so the energy information in the speech feature is also a method to analyze the speech emotion feature. The acoustic manifestations that affect the sound quality are wheezing, vibrato, choking, etc., and often appear when the speaker is emotional and difficult to suppress. In the auditory discrimination experiment of speech emotion, the change of sound quality was unanimously identified by the listeners as having a close relationship with the expression of speech emotion.

The timbre characteristic of speech is a subjective evaluation index of speech, which can be measured by formants (Hizlisoy et al., 2020). Formant frequency is a measure of acoustical characterization of sound quality. It stimulates the signal through the glottis, the frequency value at which resonance occurs in the vocal tract. Therefore, different states and different emotions have different resonance frequencies. We can analyze speech features by different sound frequencies. Voices of different emotional types in sound quality will cause different deformations of the vocal tract, which will affect the resonance peak.

#### HHT Theory

**Figure 2:**
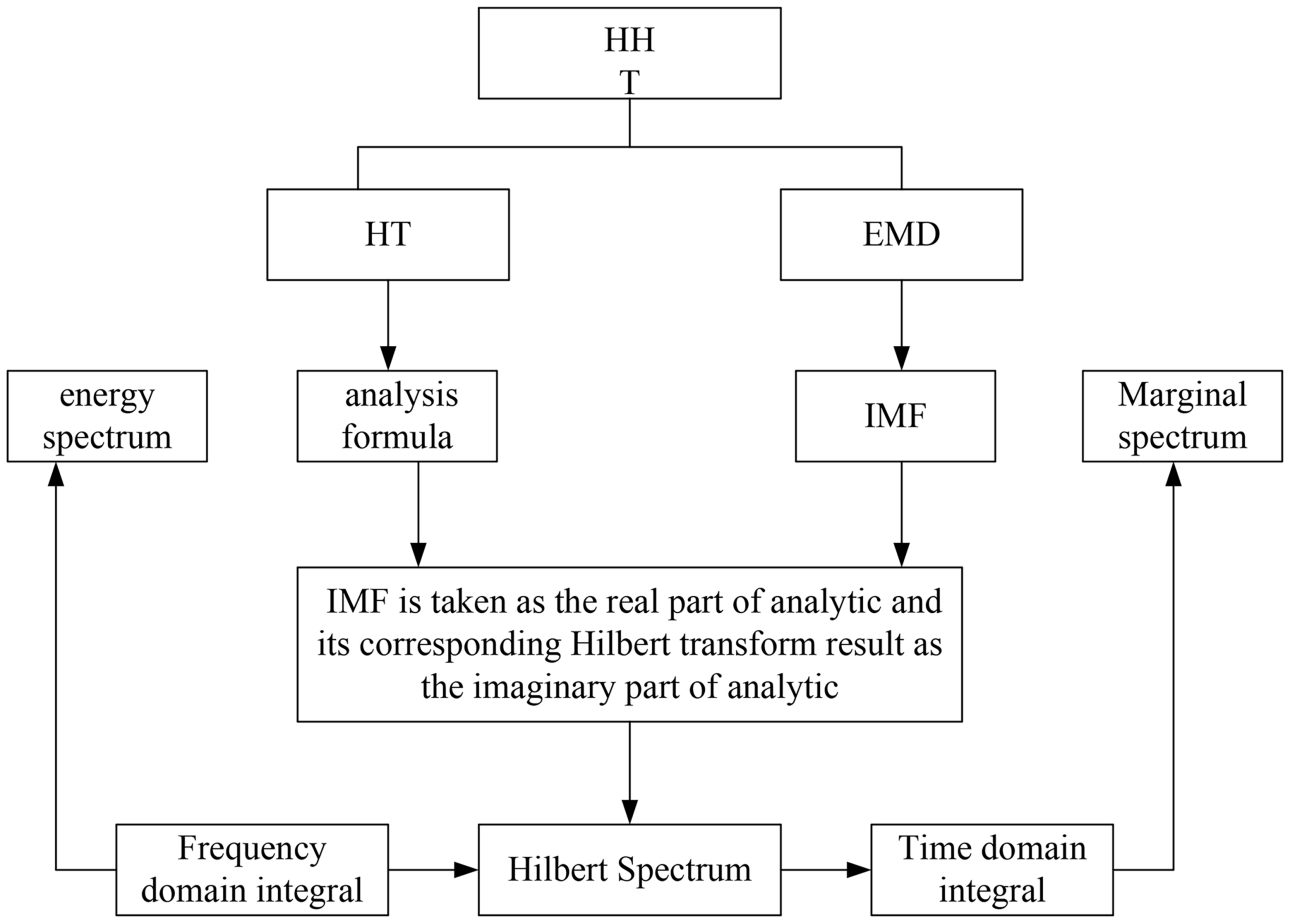
The application process of HHT theory.

The current processing idea of speech emotion recognition is still to treat it as a typical pattern recognition problem. Therefore far, almost all pattern recognition algorithms have been used (Yan et al., 2019). Using speech emotion signal processing and analysis methods, the local time-frequency characteristics of the signal can be obtained. That is, at any time, we can obtain the instantaneous frequency of the signal to better conduct emotion recognition research. HHT theory is often used in speech emotion signal analysis. The two most commonly used methods in HHT theory are: empirical mode decomposition and Hilbert transform. As shown in Figure 2, it is the application process of HHT theory.

1. Empirical Mode Decomposition is a method to directly decompose signals in the time domain (Rao, 2020). Compared with many traditional and classical methods, such as Fourier transform, wavelet transform, etc., EMD is adaptive. The basis function is not preset but is adaptively extracted from the data to be decomposed. Therefore, these basis functions can well reflect the characteristics of the original data. After the speech signal with non-linear and non-stationary characteristics is decomposed by empirical mode, some finite simple signal components can be obtained, which are called intrinsic mode components (Zhang and Min, 2020).

The screening algorithm is the core process in empirical mode decomposition. For the signal value given by the data, the difference function can be obtained. The formula for the difference function is:


(1) $$r\left(x\right)=y\left(x\right)-\left(y_{\mathrm{up}}\left(x\right)+y_{\mathrm{low}}\left(x\right)\right)/2$$

The original signal difference can be obtained through the above formula, and, then, the calculation formula of the residual amount can be obtained:


(2) $$w_{1}\left(x\right)=y\left(x\right)-s_{1}\left(x\right)$$

The above formula yields the initial signal, which repeats the screening process described above. It extracts the intrinsic state components inside the original signal. The calculation process cannot be stopped until it satisfies the residual algorithm. If their value is too large or too small, or if the inflection point in the calculation is not reached, the operation can be stopped (Sun et al., 2018). Eventually, it gets the intrinsic state components of a series. With these signal recombinations, it can get the basis function of y(x) of the original signal:


(3) $$y\left(x\right)=\overset{E}{\underset{j=1}{\sum }}s_{j}\left(x\right)+w_{e}\left(x\right)$$

E stands for the number of intrinsic modal components. They all contain the properties of mutual orthogonality and mean equal to zero.

2. Hilbert transform is a part of HHT theory. In the acquisition of the original signal, the Hilbert spectrum in the Hilbert transform can make the original signal from a three-dimensional spectrum of amplitude-frequency-time. It uses the Hilbert spectrum to obtain the instantaneous frequency and instantaneous energy value of the original signal in the corresponding period (Zhang, 2017). Through the Elbert transform, it can more conveniently process the signal with non-linear and non-stationarity, which has great advantages compared with the traditional Fourier transform method.

For a signal that satisfies the transformation conditions, the formula for defining the Hilbert transformation is:


(4) $$h\left(x\right)=\frac{1}{\pi }K\overset{+\theta }{\underset{-\theta }{\int }}\frac{r\left(\tau \right)}{x-\tau }l\tau$$

The above formula expresses the result of the Hilbert transform. It takes the variable signal as the real part and h(x) as the imaginary part, and a corresponding analytical expression w(x) can be obtained. Its formula expression is:


(5) $$w\left(x\right)=y\left(x\right)+\mathrm{uh}\left(x\right)=b\left(x\right)e^{u\mu \left(x\right)}$$

It contains the instantaneous amplitude and instantaneous phase of the analyzed signal. It can be represented by Cartesian coordinates and polar coordinates, respectively, and the expression formula is as follows:


(6) $$b\left(x\right)={\left(y^{2}\left(x\right)+c^{2}\left(x\right)\right)}^{1/2}$$

(7) $$\beta \left(x\right)=\tan^{-1}\left(c\left(x\right)/y\left(x\right)\right)$$

Finally, the derivative of the instantaneous frequency is defined as the instantaneous phase:


(8) $$\gamma \left(x\right)=\frac{l\beta \left(x\right)}{\mathrm{lx}}$$

After the instantaneous amplitude and instantaneous frequency, the expansion of the original signal after the HHT method can be expressed as:


(9) yx=Re∑j=1Wcjxui∫γjxlx

According to the above analysis, the Fourier series expansion of the original signal is obtained:


(10) yx=Re∑j=1θcjeu∫γjlx

It can be found that the cardinality of the original signal in the Fourier series expansion is infinite. They are constant over the span of the entire computation.

#### Speech Signal Preprocessing Technology

In the process of speech signal processing, due to the influence of factors such as glottal excitation and oral and nasal radiation, the average power spectrum of the speech signal drops at a speed of 6 dB/oct at the high-frequency end. Therefore, when calculating the spectrum of the speech signal, the components at the high-frequency end are relatively small, which makes it difficult to obtain the spectrum of the high-frequency part (Saurav et al., 2022). In response to the problems found above, it proposes a pre-emphasis technique to perform spectral analysis on channel parameters. By processing the high-frequency part, it calculates the signal-to-noise ratio of the signal and flattens the frequency of the signal. The pre-emphasis process is generally added after the digitization of the speech signal. It is usually a first-order digital filter:


(11) $$F\left(G\right)=1-\varepsilon g^{-1}$$

The value of μ is close to 1.

**Figure 3:**
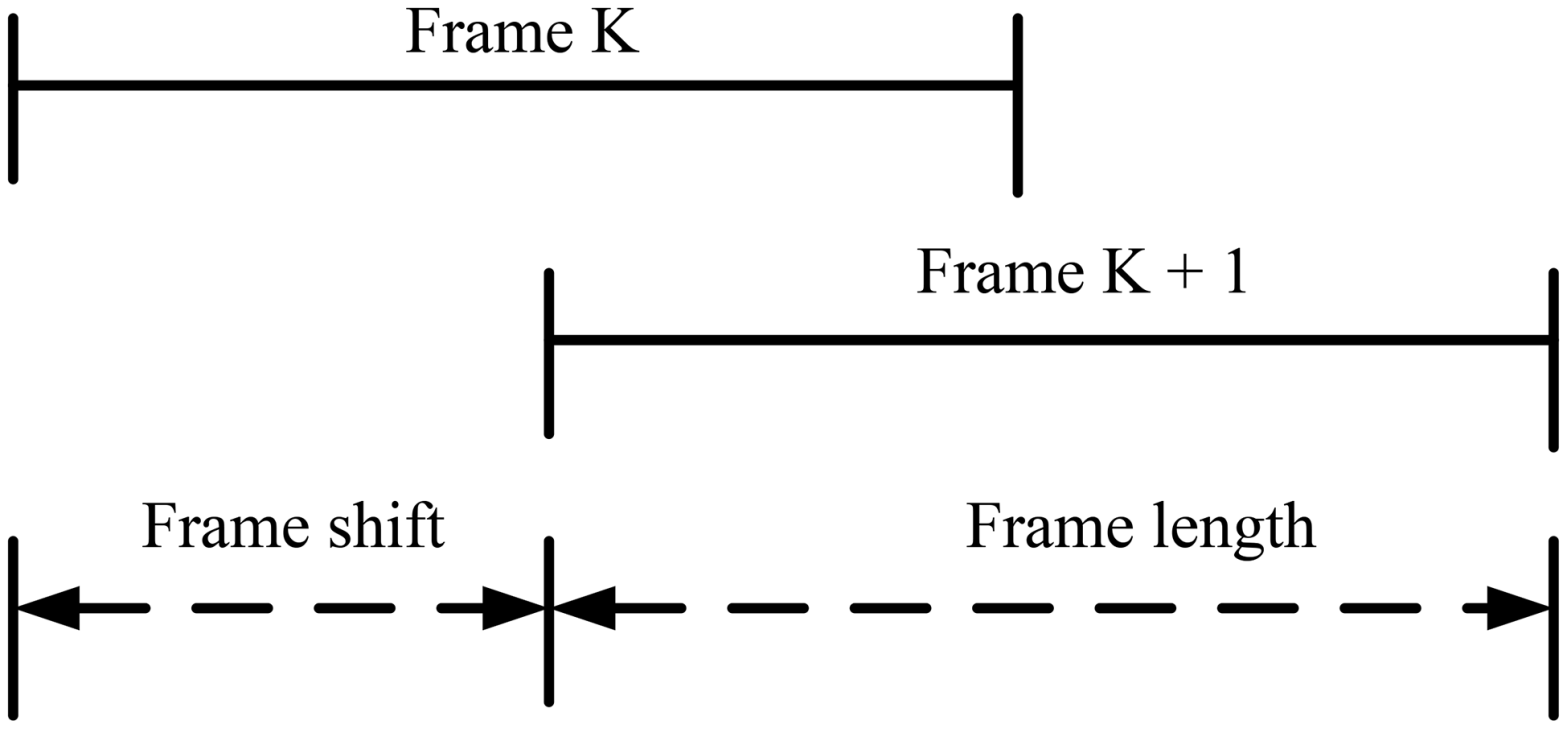
The relationship between frame length and frame shift.

The voice signal is different from other signals, so the voice signal needs to be framed. The speech signal is a non-stationary signal, and the way of processing the stationary signal cannot process the speech signal. The speech signals in the same sound range in a short time are split, as shown in Figure 3. To maintain the continuity of the split signal, it uses two methods of overlapping framing and continuous framing to preprocess the signal.

In the process of signal analysis, it performs window processing on the speech signal, and a rectangular window is often used. This is to reduce the impact of spectral leakage during framing. The expression for a rectangular window is:


(12) $$Q\left(g\right)=\{\begin{array}{c} 1,0\leq g\leq \left(G-1\right)\\ 0,others \end{array}$$

The expression in the frequency domain is:


(13) $$S_{W}\left(r^{j\propto }\right)=r^{-j\left(\frac{G-1}{2}\right)\propto }\frac{\sin\left(\frac{\propto G}{2}\right)}{\sin\left(\frac{\propto }{2}\right)}$$

Finally, end-point detection is performed on the noise that may exist in the speech signal, and it extracts useful signals in the speech signal to achieve the efficiency of speech emotion recognition. The noise that may exist in the speech signal will affect the accurate extraction of speech emotion feature parameters, which will have a certain impact on the recognition results.

#### Speech Emotion Feature Extraction Method

It uses the SVM algorithm to extract speech emotion features. It enables fast and efficient data processing across platforms, high modularity and reusability of components, plug-in support, multi-threading, support for parallel feature extraction, etc. (Liu and Wu, 2019). From the distribution of the maximum value of energy in different emotions, it can be seen intuitively that the maximum value of energy has a strong ability to distinguish emotions. It can also be obtained that the emotional discrimination of the feature is less than the maximum energy value in the dataset. This kind of feature that has nothing to do with emotion recognition has neither emotion recognition degree but also increases the unnecessary calculation amount.

It chooses the support vector machine as the classifier for speech emotion recognition. Since SVM is a linear classifier, each trained model intelligently distinguishes two types of data during the classification process (Liliana et al., 2019). In the identification process, attention should be paid to transforming the multi-class classification problem into multiple two-class classification problems. Finally, it makes decision-making decisions on multiple recognition models and then realizes the classification of multiple types of emotions. There are two kinds of common support vector machine multi-class conversion into bodies. One is a one-to-many classification method and the other we call a one-to-one classification method. One-to-many classification is to classify each emotion and other emotions into two categories. Then, it compares the recognition rate of each classification problem model, and the emotion with the highest recognition rate is the emotion we finally recognize.

This method is kind of flawed because the training set is 1:M. There is a serious imbalance in the sample size in this case. This makes this model have a high probability of affecting the prediction accuracy, so it is not very practical. In addition to the one-to-many classification method, the commonly used support vector machine multi-classification processing method also has a one-to-one classification method (Demircan and Kahramanli, 2018). The one-to-one specific method is to classify each type of emotion data in the multi-type emotion into one category and then combines each type of emotion in pairs to construct multiple and classified emotion recognition models. It finally votes on the recognition results of each model to get the final result.

### Image Emotion Recognition

The outpouring of emotion is not instantaneous but changes over time. Pleasure or anger is a process of continuous change in expression and voice, which transitions from a little emotional expression to an exaggerated emotional burst, not isolated and instantaneous. Therefore, processing dynamic information is an important way for the brain to process emotional information (Ramli et al., 2018). Because human expressions changes with the change of emotions over time, the analysis of expression is the main process of emotion recognition.

#### Expression Feature Point Extraction of DMF_MeanShift

It identifies and processes the information conveyed by the changes of people’s mouth, nose, eyes, eyebrows, and expressions in the image. The DMF_MeanShift algorithm is used to detect the image information, and it is found that the DMF_MeanShift algorithm has great advantages in the accuracy and speed of detection pairs. It can get the point distribution model formula of DMF_MeanShift algorithm:


(14) $$k_{i}=sr\left({\bar{k}}_{i}+\infty_{i}w\right)+p$$

Among them, it performs scaling, rotation, and translation weight analysis on the image and finally determines an objective function with deformation constraints and matching cost constraints:


(15) $$\mu \left(m\right)=Q\left(m\right)+\overset{n}{\underset{i=1}{\sum }}F_{i}\left(y_{i}\mathrm{;U}\right)$$

It uses the probability model to perform the optimal matching of feature points to all data obtained in the above formula and obtains the maximum likelihood estimate. When the image U is known fixedly, the detectors are independent of each other, then the formula of the obtained likelihood function is:⋈


(16) $$m\left(\mathrm{m|}{\left\{k_{i}=1\right\}}_{i=1}^{n}\mathrm{|,U}\right)⋈m\left(m\right)\;\overset{n}{\underset{i=1}{\prod }}m\left(k_{i}=1{\mathrm{|y}}_{i}\mathrm{,U}\right)$$

According to the above formula, the optimal matching point of the feature points is obtained, and the logarithm is obtained:


(17) S(m)=−ln{m(m)}

(18) $$H_{i}\left(y_{i};U\right)=-\ln\left\{m\left(k_{i}=1|y_{i},U\right)\right\}$$

### Optical Flow Motion Feature Calculation

To obtain the movement process of expression feature points over time, that is, the change process of expression, this paper uses the Lucas–Kanade optical flow method to track continuous multi-frame expression images. It captures the dynamic features of expressions that change over time. It marks image pixels. Considering the large dimension of expression features generated by the global optical flow, it cannot be directly input to the classifier for recognition. According to the sparse coding mechanism of visual cortex cells, it incorporates the idea of sparseness into the algorithm. This preserves key features and greatly reduces feature dimensionality. It is derived from the motion points of the feature points in different time periods, and we can get:


(19) U(y+fy,g+fg,x+f,x)=U(y,g,x)

When fx tends to 0, the optical flow constraint formula can be obtained:


(20) $$U_{x}r+U_{x}m+U_{t}=0$$

#### Improved Recurrent Neural Network for Dynamic Expression Recognition

**Figure 4:**
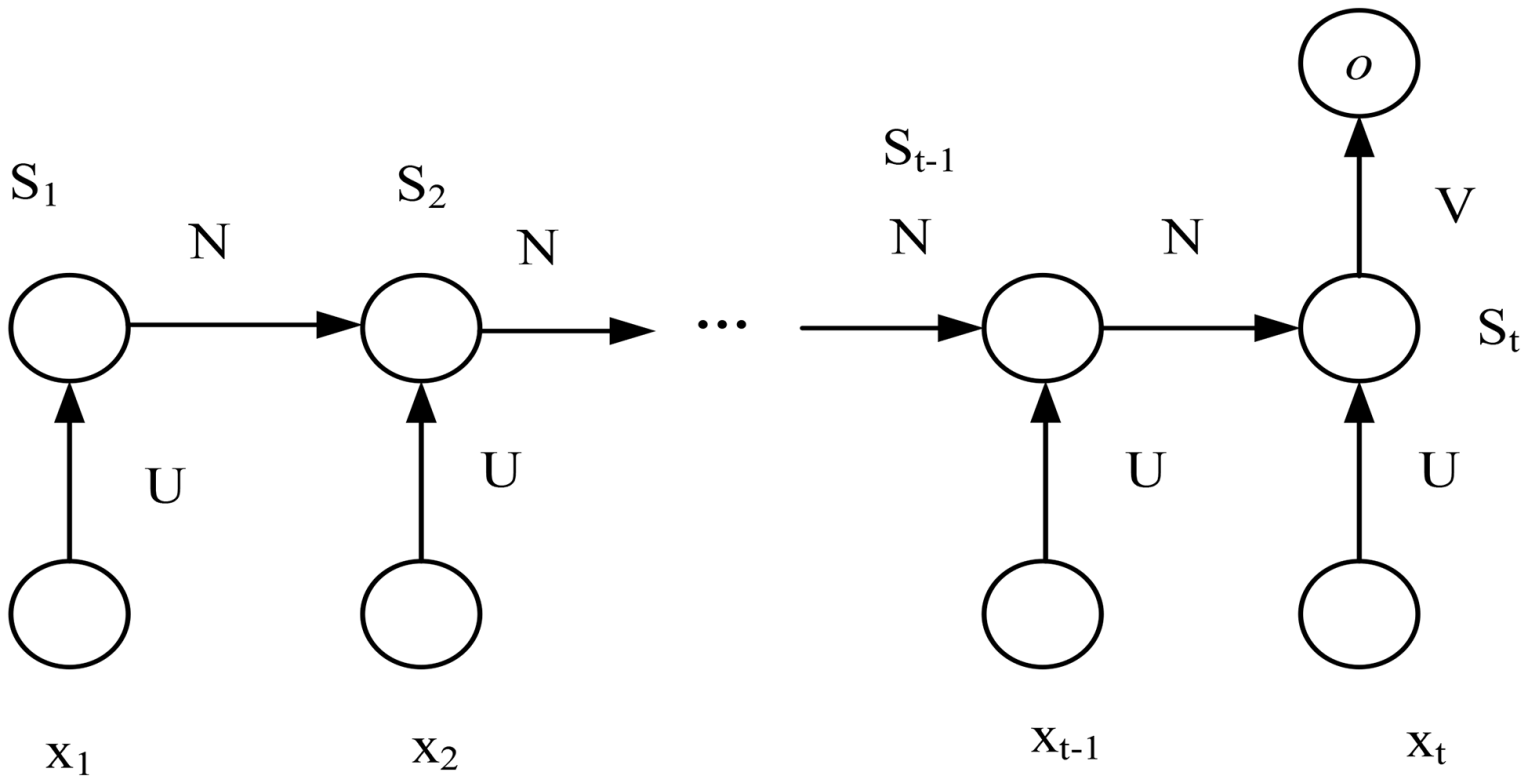
RNN model with multiple inputs and single output.

Using recurrent neural networks for facial expression recognition can make full use of the temporal features in image sequences. In the traditional RNN structure, there is an output at each step, but this is not required. Since the speech signal is continuous in the time domain, the feature information extracted by the frame only reflects the characteristics of a single frame of speech. The recurrent neural network can increase the correlation information between the previous and previous frames in the feature dimension. For example, when predicting the emotion expressed by an image sequence, it only needs to care about the output after the input of the last frame of image and does not need to know the output after the input of each frame of image. Figure 4 shows the improved multi-input single-output RNN model.

### Audio-Visual Fusion Multi-Modal Emotion Recognition System

**Figure 5:**
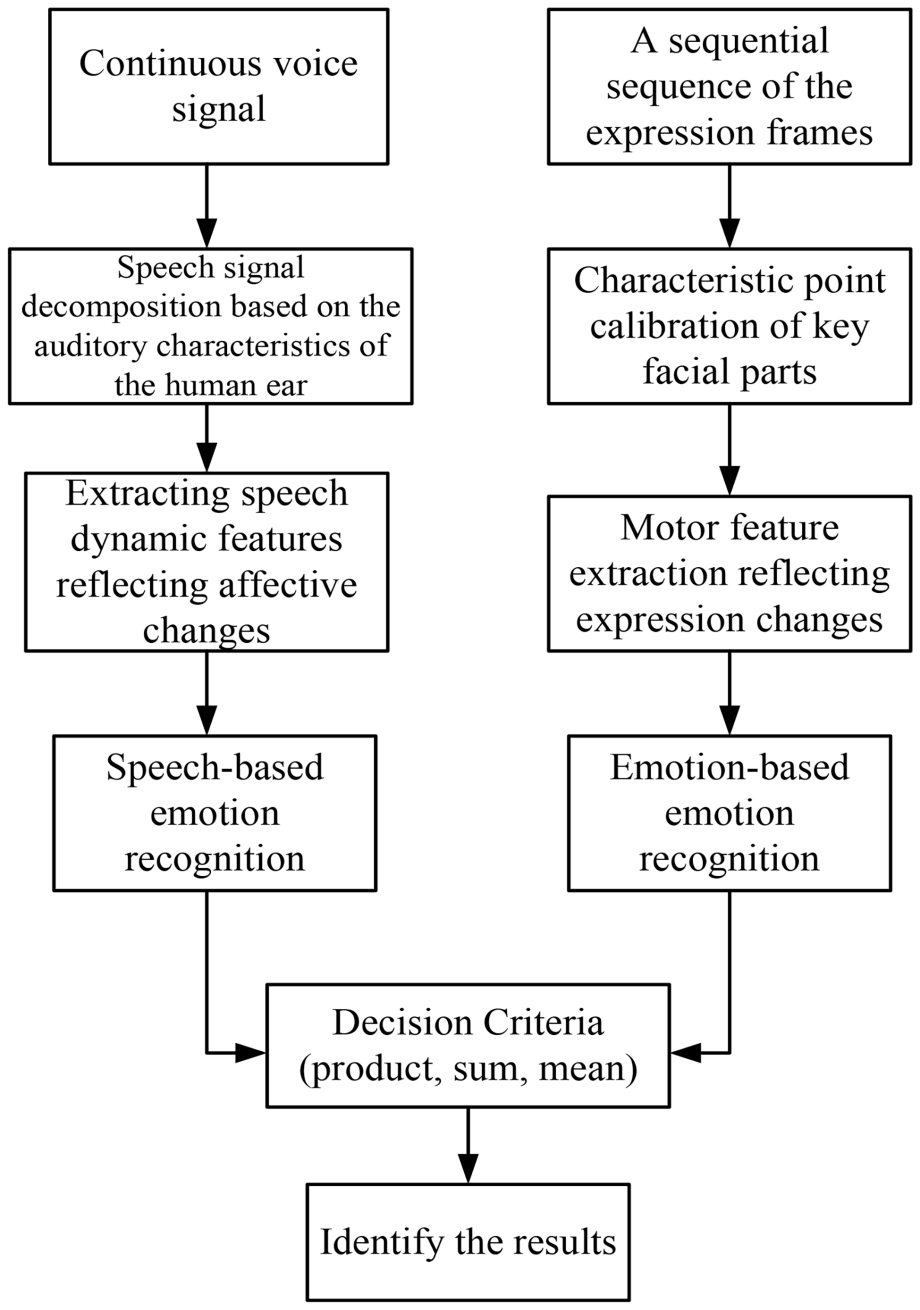
Block diagram of multi-modal emotion recognition based on speech and expression.

It constructs a multi-modal emotion recognition system integrating speech and expression, which is used in the teaching mode of English flipped classroom. Figure 5 is a block diagram of multi-modal emotion recognition based on speech and expression (Mei, 2017). It obtains speech and facial expression information from image data to build a multi-modal emotion database and conducts research from two aspects: feature layer fusion and decision layer fusion. The decision-making layer uses two RNN classifiers for speech emotion recognition and facial expression recognition, respectively, and then uses the three rules of product, sum, and mean to make decisions to obtain the final result (Lakshmi and Krithika, 2017). RNN can fully exploit the correlation features between frames to make up for the deficiency of insufficient training samples. Feature layer fusion reconstructs speech and expression features into a high-dimensional matrix and then uses RNN to identify the result.

#### Decision-Making Level Fusion

**Figure 6:**
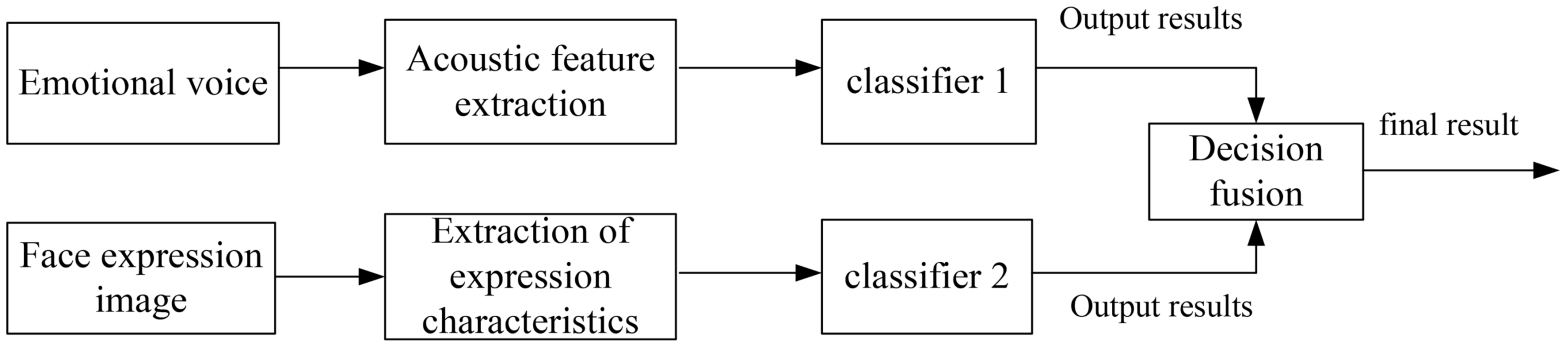
Schematic diagram of decision-making layer fusion emotion recognition.

Decision-level fusion refers to the identification experiments of single modes, respectively, and after the results are obtained, the results obtained by each classifier are fused according to certain rules to obtain the final result. In this experiment, three methods of product rule, sum rule, and mean rule are used for decision fusion. As shown in Figure 6, it is a schematic diagram of decision-making layer fusion.

The emotion of different states is classified, and the feature sets of similar types are fused according to certain rules to obtain a new probability set, and the optimal set is selected as the final recognition result.

#### Feature Layer Fusion

**Figure 7:**
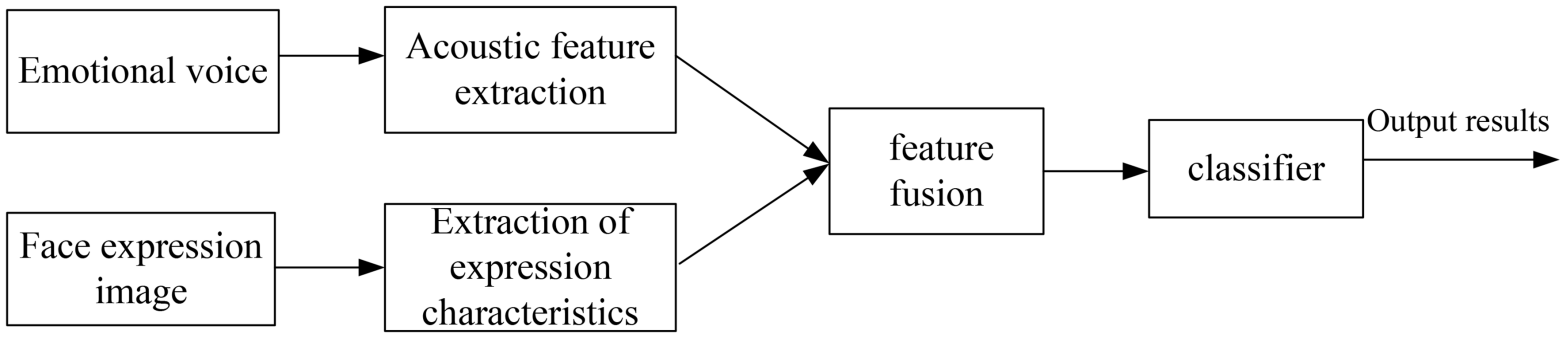
Schematic diagram of feature layer fusion emotion recognition.

Feature layer fusion refers to using a certain method to fuse facial expression features and speech emotion features into a total feature vector. It is finally input to the classifier for recognition to obtain the final result (Tang et al., 2022). Sequence forward selection is a bottom-up search method where the number of features increases one by one from zero. The construction method of the final feature set is to add new features on the basis of the existing feature set until the number of features reaches a certain value. The schematic diagram of feature layer fusion is shown in Figure 7.

Because auditory stimuli can promote visual judgment, multiple speech features and multiple expression features are fused to obtain multiple audio-visual combined vector features. The data of fusion features are shown in Table 1.

**Table 1:** Features extracted from multi-modal emotion recognition experiments.

Feature category		Statistical quantity	Dimension
phonetic feature	Subband energy	Maximum, minima, mean, variance,22-dimensional first-order difference coefficient	26
	MFCC	MFCC parameters	22
	Formant	Maximum, minima, mean, variance,	24
	Fundamental	Maximum, minima, mean, variance of the first and second derivatives	32
	Short-time energy	Energy and its first and second order derivatives	32
Expression characteristics	Outline	Optical flow of pixel points	17
	Eyebrow	Optical flow of pixel points	10
	Eye	Optical flow of pixel points	12
	Nose	Optical flow of pixel points	9
	Mouth	Optical flow of pixel points	20
Summation		204	

### Emotion Database

**Figure 8:**
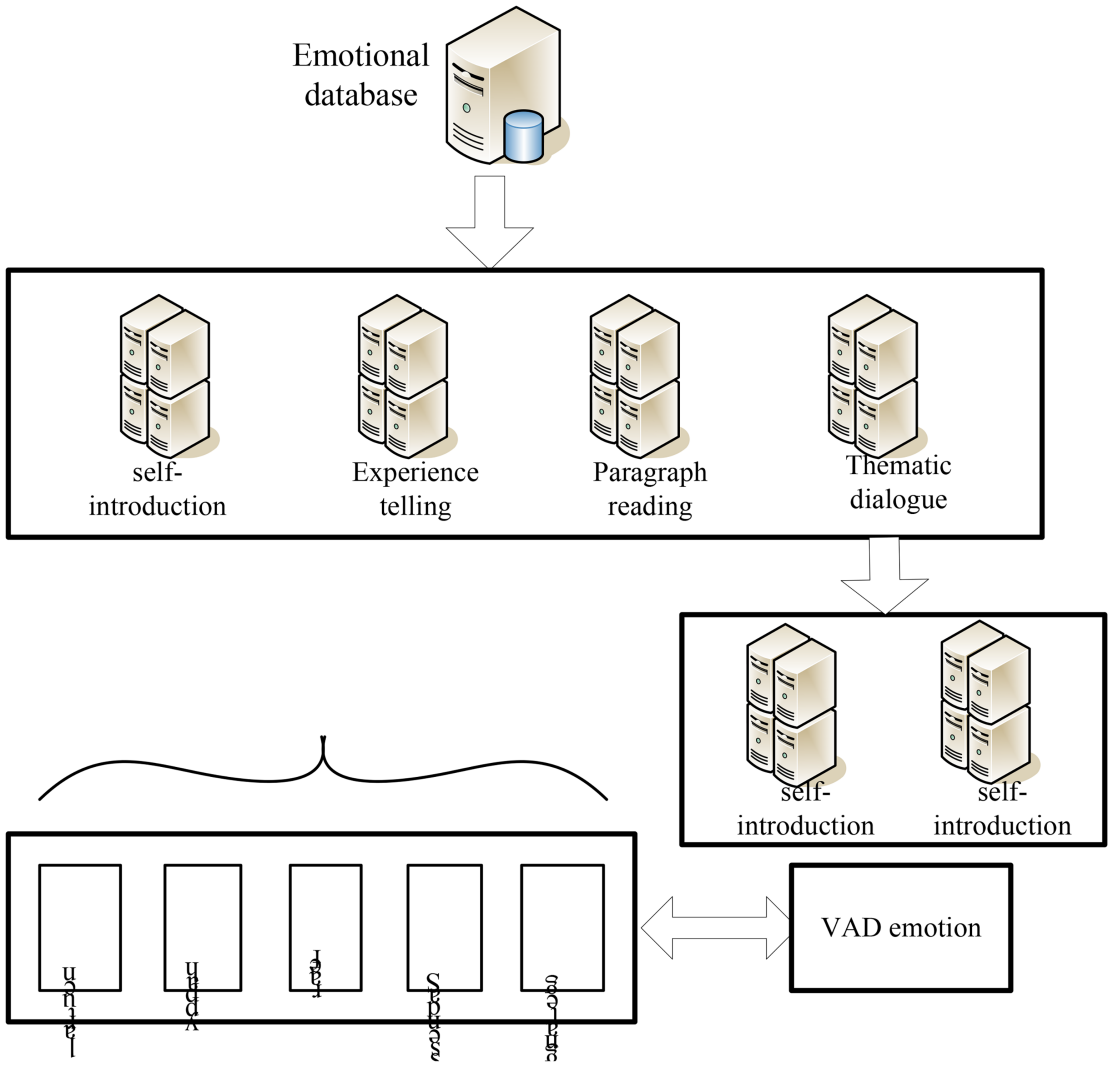
Composition of emotion database.

In the process of constructing the English flipped classroom teaching mode, it supplements the construction of a new emotion database for subsequent training and identification. It also establishes a dimension discretized emotional speech database annotation model by annotating the database. Figure 8 shows the composition of the emotion database. In the subsequent feature extraction process, it converts the speech signal into feature information that can identify emotion categories through methods such as signal preprocessing, feature extraction, and feature set selection. Finally, it trains and recognizes the annotated feature information through the support vector machine. It constructs different classification models through two different classification ideas and finally analyzes the results (Jain et al., 2022).

**Figure 9:**
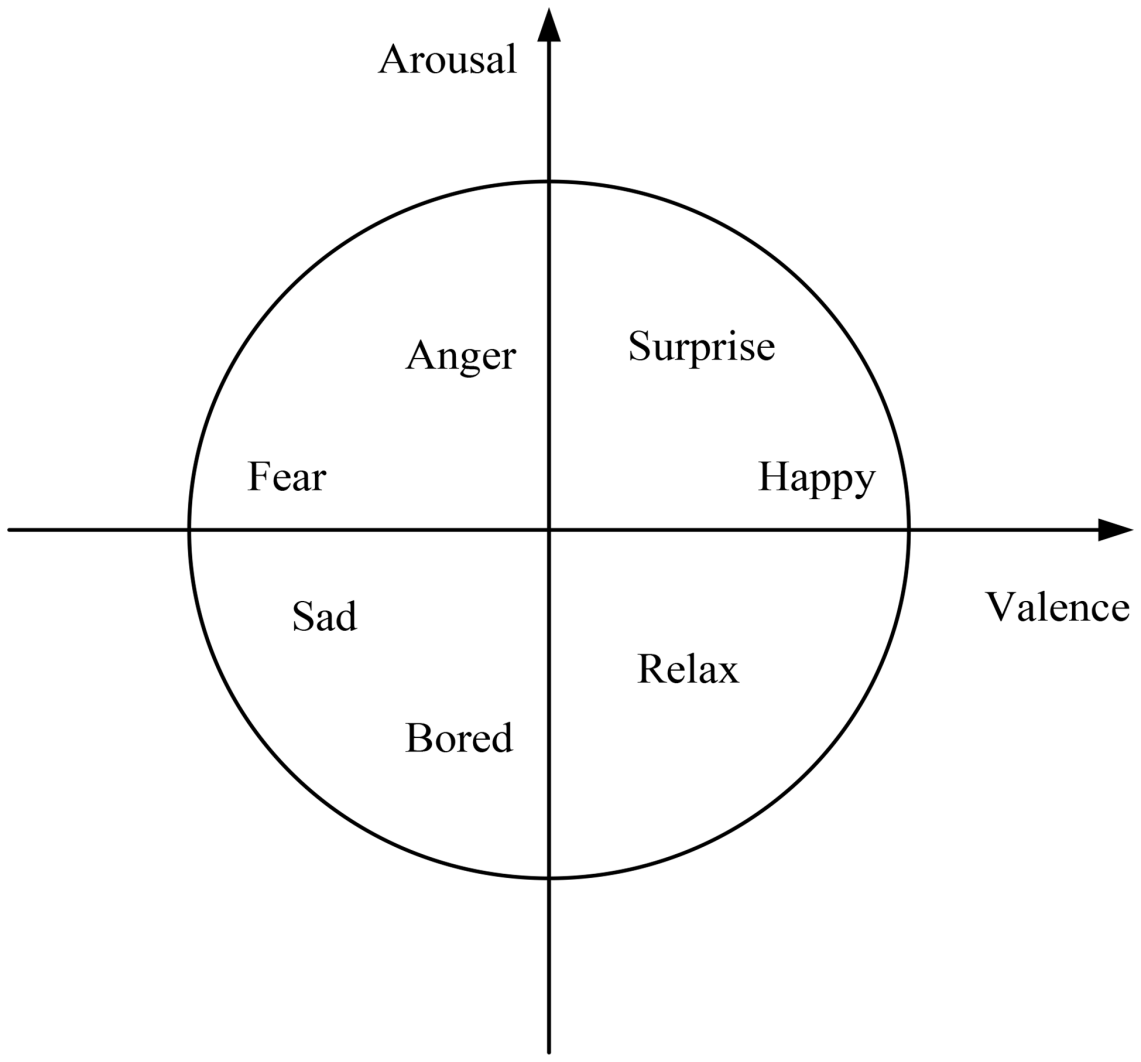
Valence-Arousal coordinate system.

After recording the corpus database, it is necessary to carry out the labeling work. To avoid major differences in the cognition of the same emotion among the annotators, the annotators should conduct a trial operation on the trial sample before the formal annotation. They compare their scoring results with standard results to reduce the negative correlation of labeling results (Singh and Dewan, 2020). The tagger first judges the basic emotion expressed by the image or audio. They select the corresponding coordinates in the coordinate system according to the distribution of the points of the basic emotions imported in advance, and then obtain their corresponding VAD values. Figure 9 is the Valence-Arousal coordinate system diagram.

The VAD values were: V (Valence) valence, A (Arousal): arousal, and D (Dominance): dominance.

Valence refers to the positive and negative affective characteristics of an individual’s affective state. The same target may have three valences for each person: positive, zero, and negative. If the individual likes the result, it is a positive valence. It has a zero value if the individual disregards its results. If the individual does not like the result, it has a negative valence. The higher the valence, the greater the motivational power. For example, if they feel happy, happy (positive affect), it is a positive valence. If they feel unhappy, unhappy (negative emotion), it is negative valence. Arousal refers to the degree of activation of the body’s energy associated with the emotional state, that is, the degree of positivity exhibited by the mind and body together. If they are energetic, it is a positive arousal. If they are dead, it is negative arousal. Dominance represents whether a person is strong or not. For example, if a person is strong, it is positive dominance. If a person is always submissive to others, it is negative dominance.

### English Flipped Classroom Teaching Mode

**Figure 10:**
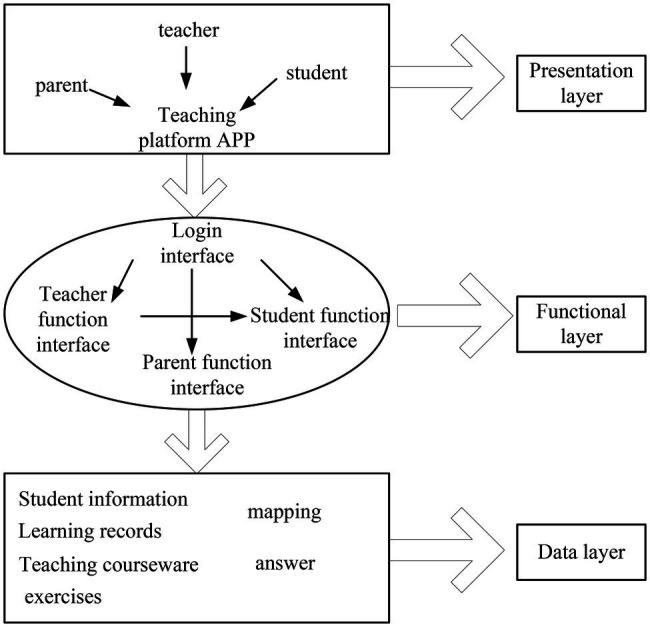
Online English flipped classroom teaching mode.

It builds an English flipped classroom teaching mode through emotion recognition technology. This paper analyzes speech emotion recognition, image emotion recognition, and audio-visual fusion emotion recognition technology and constructs an emotion database. It performs training analysis for subsequent emotion recognition. This strengthens the innovation of English flipped classroom teaching mode. As shown in Figure 10, it is an online English flipped classroom teaching mode constructed by emotion recognition technology.

It combines the English flipped classroom teaching mode of speech sentiment analysis and image sentiment analysis. The system can perform emotion recognition more clearly. It improves recognition efficiency and enhances human-computer interaction, thereby improving teaching efficiency.

## Experiment of English Flipped Classroom Teaching Mode Based on Emotion Recognition Technology

### Speech Emotion Recognition SVM Emotion Classification Simulation Experiment

It selects a primary school English flipped classroom teaching mode database to carry out the impact test of emotion recognition. The database contains more than 20,000 emotional speech samples, and the extracted features are 100-dimensional features obtained by forward sequence feature selection. Features that are not range-normalized can be difficult to distinguish meaningful terms in a classifier. Through data processing, it processes the feature data into a data structure that can be used for training the SVM classifier. It also unifies the feature range to a certain interval.

It uses the processed features for classification experiments. First, it is trained according to the one-to-one method, and, then, it is recognized according to such method, and the recognition result is obtained. We use Libsvm as the classification tool, and the multi-classification method is implemented in this way. It can directly use Libsvm to detect the recognition rate. The training data set and the test data set are used for identification.

In the process of emotion recognition for this primary school English flipped classroom teaching model, we label emotions into four categories and use these four categories for one-to-one training and identification. It is a recognition model based on the traditional SVM one-to-one method for recognition and then constructs a speech emotion recognition model based on dimension discretization. The recognition results of the two models are shown in Table 2.

**Table 2:** The recognition results of the two models.

Affective style	One-to-one identification (%)	Dimension classification identification (%)
Angry	81.4	82.2
Happy	76.9	78.7
Fear	67.8	71.1
Relax	64.4	68.7

It can be seen from Table 2 that after emotion recognition in the school’s English flipped classroom teaching mode, the final recognition rate of angry is 81.4%, the recognition rate of happy is 76.9%, the recognition rate of fear is 67.8%, the recognition rate of relax is 64.4%, and the average recognition rate is 72.6%. The recognition results of the dimension classification recognition model are 82.2%, 78.7%, 71.1%, and 68.7%, respectively, and the average recognition rate is 75.2%, which is 2.6% higher than that of the one-to-one method. After the improved emotion recognition model is classified into different dimensions, the recognition results for each emotion have a certain degree of improvement in the recognition rate.

**Figure 11:**
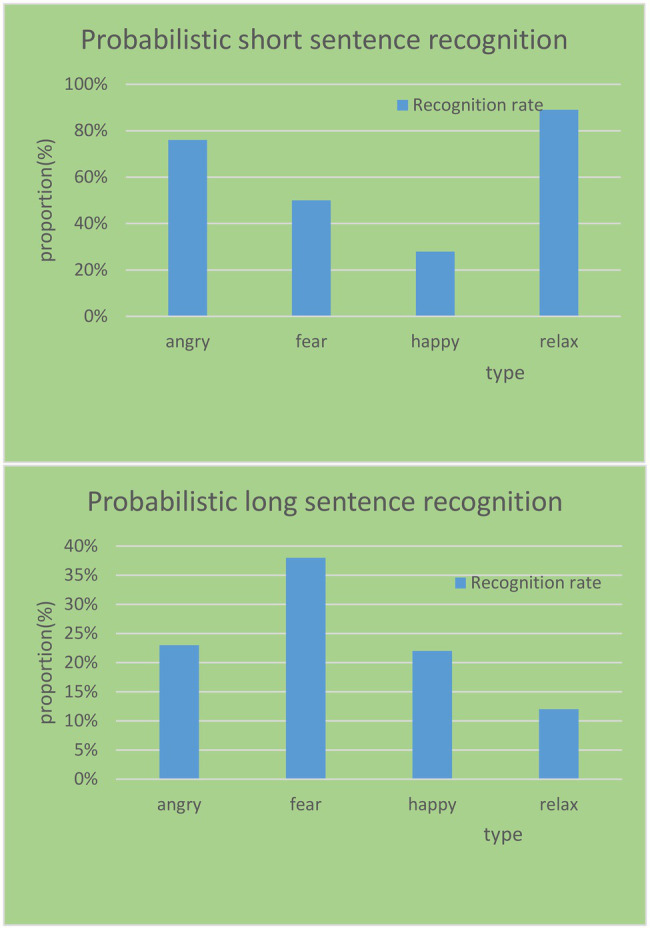
Correction rates of different emotion types on long and short sentences.

In the database, it corrects the classification of short sentences and long sentences using the HHT algorithm and obtains the correction results, as shown in Figure 11. It uses HHT, a method capable of dealing with non-linear and non-stationary signals, to extract some effective features for speech emotion recognition. It can complement traditional statistical features in speech emotion recognition, especially for a shorter corpus.

The more the semantic content in the sentence, the more interference and destruction energy distribution information in the frequency domain corresponding to the specific emotion in the speech will be. This results in the invalidation of features, which, in turn, results in a reduction in the recognition rate. Taking the sad emotion type corpus as an example, a total of five sad emotion samples were misidentified in the experiment, of which one were short sentences and four were long sentences. After fusing the features, only one short sentence was corrected; however, the four long sentences still could not be correctly identified. The most important factor is that more semantic information greatly affects the structure of the spectrum, thereby destroying the emotional expression in the spectrum. Therefore, the HHT algorithm has a better effect on the emotion recognition rate and correction rate of short sentences.

### Single-Modal and Multi-Modal Emotion Recognition Experiments

It adopts a decision-level fusion strategy based on three rules of product, sum, and mean for various emotional expressions in the English flipped classroom teaching mode for multi-modal emotion recognition. The single-modal and multi-modal emotion recognition experiments were carried out to obtain the following recognition results, as shown in Table 3.

**Table 3:** Experiment results of decision-making fusion emotion recognition.

Decision fusion strategy	Angry	Fear	Happy	Surprise	Sad	Calm	Average
Product rule identification rate of %	92.51	90.62	98.45	85.60	90.39	96.04	92.27
summation rule identification rate %	92.5	87.36	94.59	80.31	91.22	93.27	89.88
Mean-rule identification rate of %	91.45	89.57	96.38	79.86	88.32	98.52	90.68

In the decision-level fusion emotion recognition experiment based on three different strategies, the product rule has the highest recognition rate of 92.27%. Compared with the summation, the fusion method of the mean rule is found to be 2.39% and 1.59% higher, respectively. The recognition rates of happiness and calm are higher than other emotional states. The three states of anger, fear, and sadness have similar recognition rates, while the recognition rate of surprise is relatively low. Among the three fusion strategies, the product rule has the best performance, except for the surprised state, the others are above 90%, of which the happy state reaches 98%.

In the test of students’ image expression recognition in the process of English flipped classroom teaching, it selects 10 consecutive expression image sequences from each emotional segment and normalizes the images. It calibrates the feature points of each frame of the image, calculates the optical flow features between every two frames, and obtains six sets of dynamic features. It inputs each group of optical flow features to RNN in a time sequence for identification and then performs feature fusion on these six groups of dynamic images to obtain the results of the corresponding emotional features.

**Figure 12:**
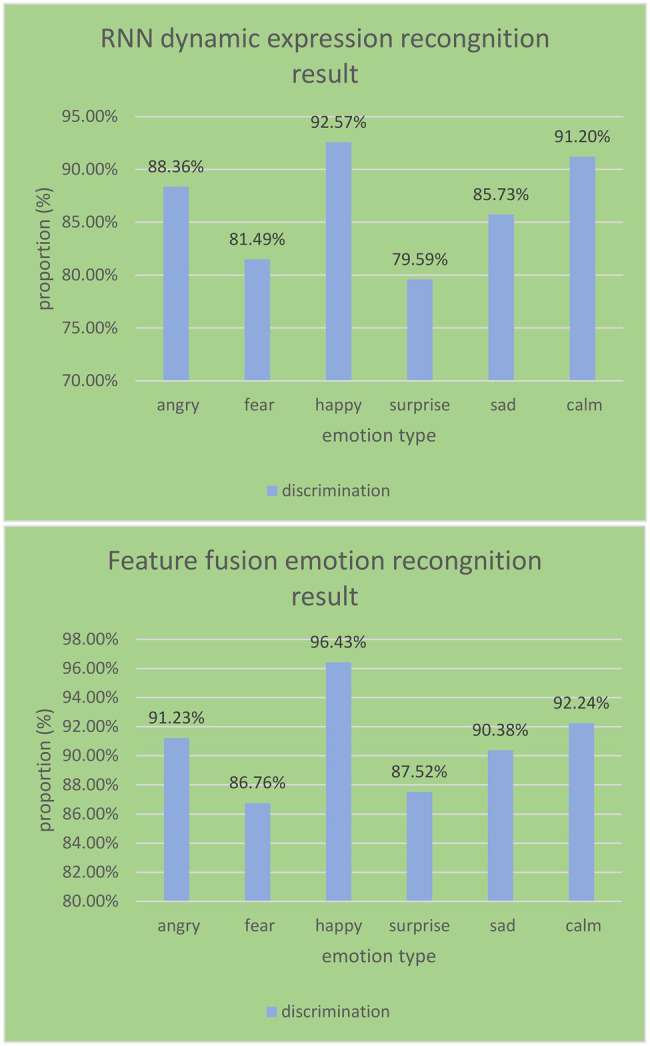
RNN emotion recognition results and feature fusion recognition results.

As shown in Figure 12, it can be seen from the RNN emotion recognition results of the English flipped classroom model that the recognition rates of happy and calm emotions both reach over 90%. They are 92.57% and 91.2%, respectively. The three emotions of sadness and fear are also above 80%, which are 88.36%, 85.73%, and 81.49%, respectively. The minimum recognition rate of surprise is only 79.59%, and the average recognition rate is 86.49%. The average recognition rate of the feature layer fusion method is 90.76%, which is 4.27% higher than the recognition rate of RNN emotion recognition. Among them, the recognition effect of a happy state is the best, reaching 96%. Angry, sad, and calm are also above 90%. The recognition rate based on the decision layer product rule is the highest, which reaches 92.27%. The feature layer fusion method is slightly lower than the decision layer fusion method, which shows that the use of different fusion rules has a certain impact on the recognition results.

Comparing the recognition performance of single-modal and multi-modal recognition techniques, it obtains the recognition results under different modalities.

**Figure 13:**
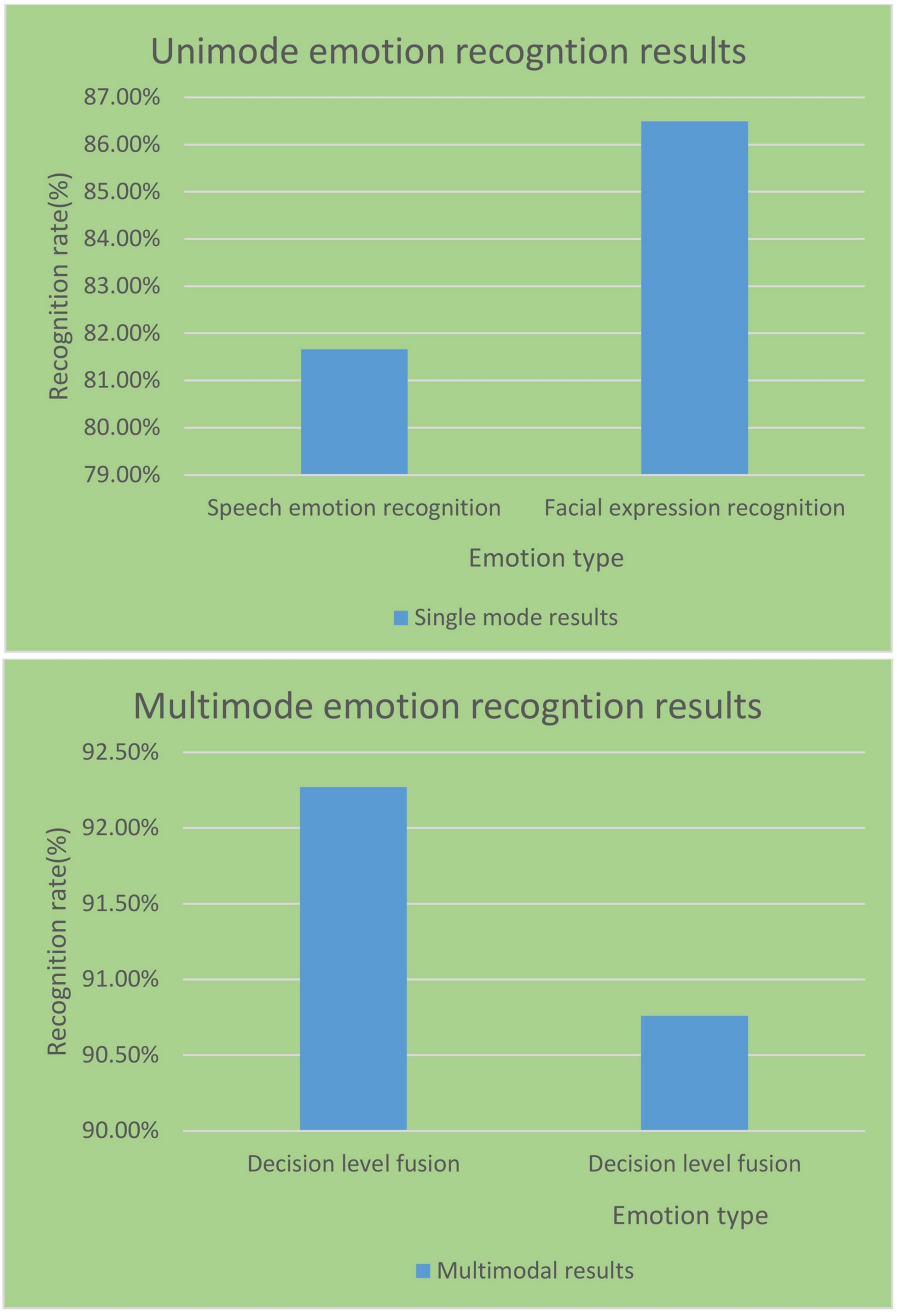
Recognition performance of single-modal and multi-modal recognition techniques.

As shown in Figure 13, the average recognition rate of expression recognition in the single-modal and multi-modal emotion recognition experiments in the English reversal classroom reached 86.49%. It is 4.83% higher than the average recognition rate of speech emotion recognition. This shows that facial expression images contain more easily distinguishable emotional features than speech signals. It can be obtained that the effect of multi-modal emotion recognition is better than that of a single modality. The emotion recognition of multi-modal information fusion has better recognition performance and is more suitable for the school’s English reversal classroom model.

### The Effect of English Flipped Classroom Teaching Mode Based on Emotion Recognition Technology

It tests and compares the performance of some students in the junior high school before and after the emotion recognition technology in the English flipped classroom teaching mode. According to the test results, the effect of the English flipped classroom teaching mode based on emotion recognition technology is inferred, and the data in Table 4 are obtained.

**Table 4:** Comparison of student achievement changes before and after identification.

Test	Group	Number	Average	Number of above 120	Number of 100–120
Before identification	1	69	90.89	3	27
	2	68	91.44	2	28
After identification	1	69	94.47	6	34
	2	68	92.76	2	30

It can be seen from Table 4 that the average scores of the two experimental groups before the experiment are lower than the scores of the experimental group after emotion recognition. The average scores of the first two groups in emotion recognition were 90.89 and 91.44, respectively. Among them, the number of students with scores above 120 points is 3 and 2. It has 27 and 28 students, respectively, with scores between 100 and 120. After emotion recognition, all data have a clear improvement. Among them, a total of eight boos scored more than 120 points. Scores between 100 and 120 increased by seven and two, respectively. This shows that after a series of emotion recognition technologies, the English flipped classroom teaching mode constructed has obtained unexpected results. Most of the students changed their negative views on the flipped classroom and raised their awareness of the flipped classroom. Understanding this model can help improve classroom efficiency and effectively alleviate the problem of boring classrooms.

## Discussion

This paper studies the influence of emotion recognition technology in the teaching mode of English flipped classroom. According to the existing problems, it constructs and optimizes the English flipped classroom teaching mode through the targeted research of speech emotion recognition, image emotion recognition, and audition fusion emotion recognition technology. It combines speech emotion recognition SVM emotion classification simulation experiment, and single-modal and multi-modal emotion recognition experiments to perform emotion recognition analysis on different decision-making layers and feature layers. It summarizes and analyzes the effect of students’ learning after the English flipped classroom teaching mode based on emotion recognition technology. It was found that reasonable and effective emotion recognition technology is beneficial to improve classroom efficiency and improve students’ enthusiasm for learning.

## Conclusion

In this paper, the empirical mode decomposition in HHT theory algorithm and the application of Hillport transform in speech emotion signal recognition are firstly discussed. It uses SVM algorithm to extract speech emotional features and performs information processing and speech feature extraction across platforms. Then, it uses DMF_MeanShift’s expression feature point extraction method and optical flow motion feature calculation to recognize image emotion. It combines DNN for emotional feature recognition of dynamic images and finally combines audio-visual fusion multi-modal emotion recognition system. It obtains emotion information from the multi-modal emotion recognition block diagram of speech and expression. It studies two aspects of feature-level fusion and decision-level fusion to identify emotion types. In the practical application of the English flipped classroom teaching mode, it conducts SVM emotion classification simulation experiments for speech emotion recognition, as well as single-modal and multi-modal emotion recognition experiments. The test results show that after different dimensions of emotion recognition, the emotion recognition rate is effectively improved. The emotion recognition and recognition performance of multi-modal information fusion is better, and it is more suitable for the school’s English reversal classroom model. This paper provides a detailed analysis of emotion recognition technology. When applied to the English flipped classroom teaching mode, it brings far-reaching significance to the transformation and innovation of teaching methods.

## Data Availability Statement

The original contributions presented in the study are included in the article/supplementary material, further inquiries can be directed to the corresponding author.

## Author Contributions

The author confirms being the sole contributor of this work and has approved it for publication.

## Conflict of Interest

The author declares that the research was conducted in the absence of any commercial or financial relationships that could be construed as a potential conflict of interest.

## Publisher’s Note

All claims expressed in this article are solely those of the authors and do not necessarily represent those of their affiliated organizations, or those of the publisher, the editors and the reviewers. Any product that may be evaluated in this article, or claim that may be made by its manufacturer, is not guaranteed or endorsed by the publisher.
